# Subtyping of sarcomas based on pathway enrichment scores in bulk and single cell transcriptomes

**DOI:** 10.1186/s12967-022-03248-3

**Published:** 2022-01-29

**Authors:** Shengwei Li, Qian Liu, Haiying Zhou, Hui Lu, Xiaosheng Wang

**Affiliations:** 1 Biomedical Informatics Research Lab, School of Basic Medicine and Clinical Pharmacy, China Pharmaceutical University, Nanjing, 211198 China; 2 Cancer Genomics Research Center, School of Basic Medicine and Clinical Pharmacy, China Pharmaceutical University, Nanjing, 211198 China; 3 Big Data Research Institute, China Pharmaceutical University, Nanjing, 211198 China; 4 Department of Orthopedics, The First Affiliated Hospital, College of Medicine, Zhejiang University, Hangzhou, 310003 China; 5 Alibaba-Zhejiang University Joint Research Center of Future Digital Healthcare, Hangzhou, China

**Keywords:** Sarcoma, Subtyping, Clustering analysis, Tumor microenvironment, Immune signatures, Genomic instability

## Abstract

**Background:**

Sarcomas are highly heterogeneous in molecular, pathologic, and clinical features. However, a classification of sarcomas by integrating different types of pathways remains mostly unexplored.

**Methods:**

We performed hierarchical clustering analysis of sarcomas based on the enrichment scores of 14 pathways involved in immune, stromal, DNA damage repair (DDR), and oncogenic signatures in three bulk tumor transcriptome datasets.

**Results:**

Consistently in the three datasets, sarcomas were classified into three subtypes: Immune Class (Imm-C), Stromal Class (Str-C), and DDR Class (DDR-C). Imm-C had the strongest anti-tumor immune signatures and the lowest intratumor heterogeneity (ITH); Str-C showed the strongest stromal signatures, the highest genomic stability and global methylation levels, and the lowest proliferation potential; DDR-C had the highest DDR activity, expression of the cell cycle pathway, tumor purity, stemness scores, proliferation potential, and ITH, the most frequent *TP53* mutations, and the worst survival. We further validated the stability and reliability of our classification method by analyzing a single cell RNA-Seq (scRNA-seq) dataset. Based on the expression levels of five genes in the pathways of T cell receptor signaling, cell cycle, mismatch repair, focal adhesion, and calcium signaling, we built a linear risk scoring model (ICMScore) for sarcomas. We demonstrated that ICMScore was an adverse prognostic factor for sarcomas and many other cancers.

**Conclusions:**

Our classification method provides novel insights into tumor biology and clinical implications for sarcomas.

**Supplementary Information:**

The online version contains supplementary material available at 10.1186/s12967-022-03248-3.

## Background

Sarcomas, a type of cancer that develops in the bones and soft tissues, are highly heterogeneous in pathologic and clinical features [1]. Usually, bone sarcomas develop more frequently in children while soft tissue sarcomas occur more frequently in adults. Soft tissue sarcomas harbor at least tens of malignant histological types and subtypes [2]. However, even though the lesions of sarcomas are broadly distributed throughout the body, the molecular characterization of sarcomas has potential for their diagnosis and management [2]. By multi-omics analysis of somatic mutations, copy number alterations (CNAs), methylation levels, and RNA and protein expression, The Cancer Genome Atlas (TCGA) Research Network [1] comprehensively characterized the molecular landscape of six types of soft tissue sarcomas, including dedifferentiated liposarcoma (DDLPS), leiomyosarcoma (LMS), undifferentiated pleomorphic sarcoma (UPS), myxofibrosarcoma (MFS), malignant peripheral nerve sheath tumor (MPNST), and synovial sarcoma (SS). This study demonstrated two key findings for these adult soft tissue sarcomas: (1) CNAs are predominant over somatic mutations; and (2) molecular subtypes and the tumor immune microenvironment are highly associated with clinical outcomes. Besides, many studies have performed molecular classification of sarcomas based on genomic profiling. For example, Kim et al. classified complex karyotype sarcomas into three subtypes based on their *CDK4* and *RB1*-associated CNAs [3]. Lee et al. classified SS, LMS, and malignant fibrous histiocytoma (MFH) into four classes based on expression profiling of 833 genes [4]. Koelsche et al. developed an algorithm to classify sarcomas based on DNA methylation profiling [5]. Gibault et al. identified five subtypes of soft tissue sarcomas with complex genomics by clustering analysis of transcriptome data [6].

Despite these previous molecular classification studies for sarcomas [1, 3–5], a classification of sarcomas by integrating different types of pathways remains mostly unexplored. Abundant evidence has shown that sarcomas are highly heterogenous in the immune and stromal microenvironment [1, 7], genomic instability [1, 8], and oncogenic pathways [9]. Thus, a classification of sarcomas based on these features of pathways has the potential to provide new insights into the heterogeneity of sarcomas. To this end, we implemented unsupervised clustering of sarcomas based on the enrichment scores of 14 pathways. These pathways were associated with immune regulation (natural killer cell-mediated cytotoxicity, antigen processing and presentation, T cell receptor signaling, B cell receptor signaling, and JAK-STAT signaling), stromal signatures (ECM-receptor interaction, focal adhesion, adherens junction, and calcium signaling), DNA damage repair (DDR) (mismatch repair and homologous recombination), and oncogenic signatures (TGF-β signaling, Wnt signaling, and cell cycle). Because the pathway enrichment score integrates the expression levels of a set of genes into a single value, the pathway enrichment-based clustering is likely to exhibit higher stability and robustness than the gene expression profiles-based clustering. In addition, the pathway enrichment-based clustering may result in more straightforward and explainable results relevant to the subtyping of cancers than the gene expression profiles-based clustering. In fact, the pathway (or gene set) enrichment-based clustering method has been employed in many recent studies and shown its advantages over the gene expression profiles-based clustering method [10–13]. By the pathway-based clustering analyses, we identified three subtypes of sarcomas, consistently in three different datasets. We further provided a comprehensive portrait of the molecular and clinical characteristics of these sarcoma subtypes. Finally, we validated our methods and results in a single cell RNA-Seq (scRNA-seq) dataset. Our classification method may furnish new insights into the cancer biology of sarcomas and clinical implications for the management of this disease.

## Methods

### Datasets

We obtained transcriptomic and clinical data for TCGA-SARC from the genomic data commons (GDC) data portal (https://portal.gdc.cancer.gov/) and GSE30929 and GSE71121 from the NCBI gene expression omnibus (GEO) (https://www.ncbi.nlm.nih.gov/geo/). From the GDC data portal, we also obtained the profiles of somatic mutations (“maf” files), SCNAs (“SNP6” files), protein expression (level 3), and DNA methylation (HM450) for TCGA-SARC and transcriptomic (level 3 and RSEM normalized) and clinical data for other 29 cancer types. In addition, we downloaded a single-cell RNA sequencing (scRNA-seq) dataset (GSE131309) for sarcomas from the GEO. All gene expression values (RSEM normalized) were added 1 and then log2-transformed before further analyses. A summary of these datasets is shown in Additional file 1: Table S1.

### Gene-set enrichment analysis

We calculated the enrichment scores of pathways, immune signatures, or biological processes in a tumor sample by single-sample gene-set enrichment analysis (ssGSEA) [14] based on the expression levels of its related genes (pathway genes or marker genes). The ssGSEA is an extension of the GSEA method, which outputs the enrichment scores of the input gene sets in different samples by inputting an expression matrix and a list of gene sets. We presented the pathways, immune signatures, and biological processes and their related genes in Additional file 2: Table S2. In addition, we identified KEGG pathways significantly associated with a gene set using the GSEA tool [15].

### Clustering analysis

We used a clustering method to identify sarcoma subtypes based on the enrichment scores of 14 pathways. The clustering method we used is hierarchical clustering algorithm, which is an unsupervised machine learning algorithm that determines the similarity between data points in each category by calculating the distance between them and all data points; the smaller the distance, the higher the similarity, and combining the two data points or categories with the closest distance to generate a clustering tree. The 14 pathways were involved in immune regulation, stromal signatures, DDR, and oncogenic signatures. We performed the hierarchical clustering in TCGA-SARC, GSE30929, and GSE71121, respectively, by using the R package “hclust”.

### Calculation of immune score, stromal score, tumor purity, TMB, HRD, ITH, and SCNA

We calculated the immune score, stromal score, and tumor purity for each tumor sample by the ESTIMATE algorithm [16]. The immune score quantifies the immune infiltration level in the tumor microenvironment (TME), while the stromal score quantifies the stromal content in the TME. Tumor purity represents the proportion of tumor cells in the bulk tumor. We defined TMB as the total number of non-synonymous somatic mutations in the tumor. The Homologous recombination deficiency (HRD) scores of TCGA-SARC tumors were obtained from the publication by Knijnenburg et al. [17]. We scored intratumor heterogeneity (ITH) scores by the DEPTH algorithm [18], which evaluates ITH at the mRNA level. The R package “DEPTH” was used to calculate ITH scores with the input of gene expression profiles in tumor and/or normal tissues. We used GISTIC2 [19] to calculate arm- and focal-level SCNAs and G-scores with the input of “SNP6” files.

### Construction of the prognostic risk scoring model

To build the gene expression-based linear risk scoring model (ICMScore) for evaluating the prognostic risk of sarcomas, we first identified seven of the 14 pathways using the Cox proportional hazards model by Lasso based on their enrichment scores in TCGA-SARC. The seven pathways included T cell receptor signaling, focal adhesion, adherens junction, Wnt signaling, calcium signaling, cell cycle, and mismatch repair. In each of the seven pathways, we identified the genes having strong expression correlations with the ssGSEA scores of the pathway (Spearman correlation *ρ* > 0.5). A total of 26 genes were identified, from which we selected 18 genes using the Cox proportional hazards model by Lasso based on their expression levels. The 18 genes were included in five pathways: T cell receptor signaling (*CD40LG*), focal adhesion (*FLT4* and *ITGA1*), calcium signaling (*ATP2B4*, *ADCY2*, and *FGF7*), cell cycle (*BUB1B*, *MCM4*, *CDC25A*, *CDK2*, *MCM6*, *RBL1*, and *TFDP1*), and mismatch repair (*EXO1*, *RFC5*, *MSH2*, *RPA3*, and *POLD2*). Finally, we selected five genes (*CD40LG*, *CDC25A*, *MSH2*, *FLT4*, and *ADCY2*) from the 18 genes, which were included in five different pathways and had the smallest *P*-values in the univariate Cox proportional hazards model based on gene expression levels among all genes in the same pathway. Using the five genes as independent variables, we built the linear risk scoring model (ICMScore) as follows:

$$ ICMScore = \sum\limits_{i = 1}^{5} {\beta i \times \exp (Gi)} , $$

where *G*_*i*_ represents one of the five genes and exp(*G*_*i*_) the expression level of *G*_*i*_ in the tumor; *β*_*i*_ is the regression *β* coefficient for *G*_*i*_ in its univariate Cox proportional hazards model. The function “cv.glmnet” in the R package “glmnet” was utilized for the variable selection by Lasso in the Cox proportional hazards model, and the function “coxph” in the R package “survival” was used for the univariate and multivariable Cox regression analyses.

### Analysis of scRNA-seq data

The scRNA-seq (SMART-seq2 [20]) dataset (GSE131309) for sarcomas was gene expression profiles in 6951 single cells from 12 human SyS tumors, which contained 4371 malignant cells and 2580 non-malignant cells. We used the tSNE algorithm [21] to cluster malignant cells and non-malignant cells, respectively. t-SNE produces a single map to demonstrate structure at many different scales, particularly useful for high-dimensional data [21].

### Statistical analysis

In comparisons of two classes of normally distributed data, including gene expression levels, protein expression levels, and the ratios of immune-stimulatory to immune-inhibitory signatures, we used two-tailed Student’s *t* test. In comparisons of two classes of data that were not normally distributed, we used the one-tailed Mann–Whitney *U* test. In comparisons of three classes of data, if they were normally distributed, we used the one-way ANOVA test, otherwise, we used the Kruskal–Wallis (K–W) test. When analyzing contingency tables, we utilized the Fisher’s exact test. We used the Benjamini–Hochberg method [22] to calculate FDR for adjusting for multiple tests. We used Kaplan–Meier curves to compare the survival (OS, DFS, and MFS) time between different groups and the log-rank test to evaluate the significance of survival time differences.

## Results

### Identification of sarcoma subtypes based on pathway scores

**Fig. 1:**
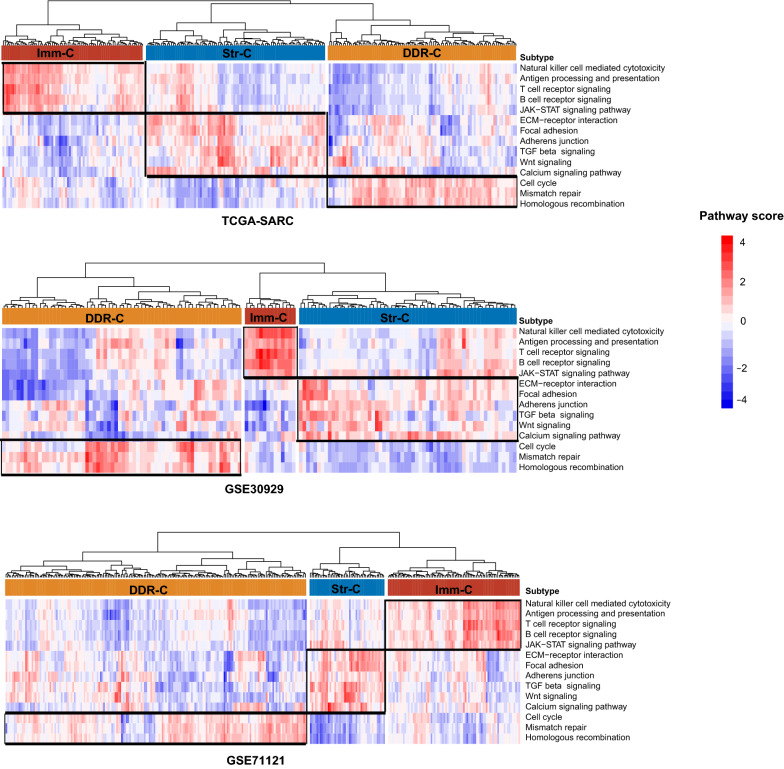
Identification of sarcoma subtypes based on pathway enrichment scores. Hierarchical clustering identifying three sarcoma subtypes: Immune Class (Imm-C), Stromal Class (Str-C), and DDR Class (DDR-C), based on the enrichment scores of 14 pathways in three different datasets (TCGA-SARC, GSE30929, and GSE71121). The pathway enrichment scores were calculated by ssGSEA [28]. The 14 pathways are involved in immune, stromal, DNA damage repair, or oncogenic signatures. DDR, DNA damage repair

We quantified the activity of a pathway in a tumor sample by the single-sample gene set enrichment analysis (ssGSEA) [14]. Based on the ssGSEA scores of the 14 pathways involved in immune, stromal, DNA damage repair, or oncogenic signatures, we hierarchically clustered sarcomas in three different datasets, respectively. The three datasets included TCGA-SARC [1], GSE30929 [23], and GSE71121 [24], which encompassed 259, 140, and 312 tumor samples, respectively. Consistently in the three datasets, sarcomas were clearly classified into three subtypes: Immune Class (Imm-C), Stromal Class (Str-C), and DDR Class (DDR-C) (Fig. 1). Among the three subtypes, Imm-C displayed the strongest immune signatures, Str-C showed the strongest stromal signatures, and DDR-C had the highest DDR activity. Meanwhile, Str-C exhibited the highest activity of the TGF-β and Wnt signaling pathways (Fig. 1). This is consistent with the prominent roles of both pathways in the activation of stromal cells [25]. DDR-C most highly expressed the cell cycle pathway. It is justified since elevated cell cycle activity must promote the DDR activity in tumors [26].

### Comparisons of immune, stromal, and DDR signatures among the sarcoma subtypes

**Fig. 2:**
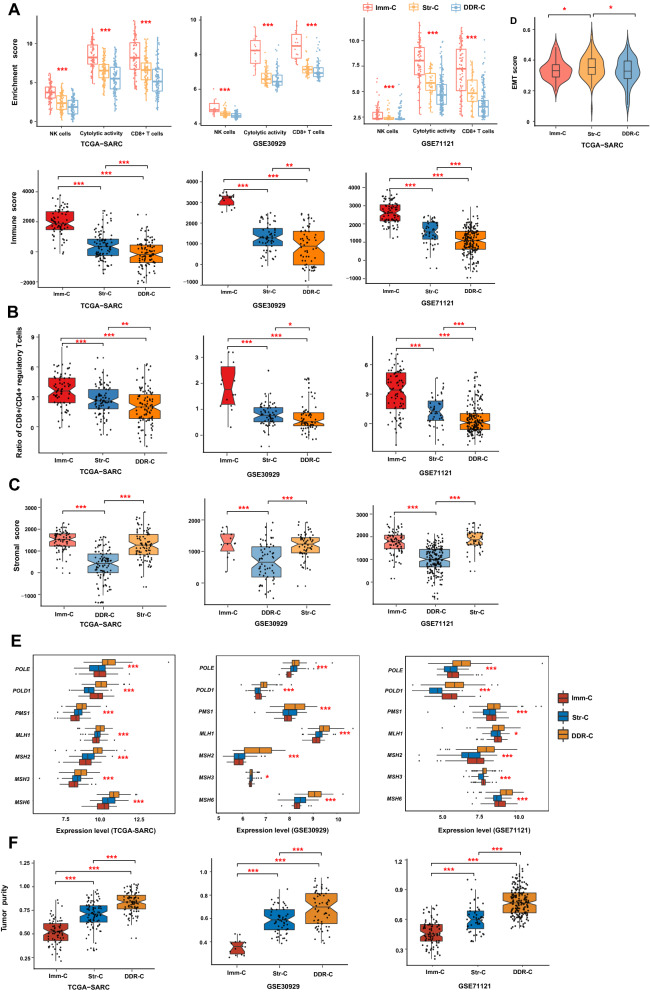
Comparisons of immune, stromal and DDR signatures among the sarcoma subtypes. Comparisons of immune signature scores (**A**), the ratios of immune-stimulatory to immune-inhibitory signatures (CD8 + /CD4 + regulatory T cells) (**B**), stromal scores (**C**), EMT scores (**D**), the expression levels of seven DDR genes (**E**), and tumor purity (**F**) among the sarcoma subtypes. The immune scores, stomal scores, and tumor purity were evaluated by ESTIMATE [16]. The EMT scores are the ssGSEA scores [28] of its marker genes. The one-tailed Mann–Whitney *U* test or two-tailed Student’s *t* test *P*-values are shown. EMT: epithelial-to-mesenchymal transition. *P < 0.05, **P < 0.01, ***P < 0.001

We compared the enrichment scores of CD8 + T cells, NK cells, and immune cytolytic activity, which were ssGSEA scores of their marker gene sets, among the sarcoma subtypes. All these immune signatures exhibited consistent expression patterns among these subtypes: Imm-C > Str-C > DDR-C, in all three datasets (one-tailed Mann–Whitney *U* test, *P* < 0.01) (Fig. 2A). In addition, we compared immune scores, which were evaluated by the ESTIMATE algorithm [16], among the sarcoma subtypes. Likewise, the immune scores followed the pattern: Imm-C > Str-C > DDR-C, in all three datasets (one-tailed Mann–Whitney *U* test, *P* < 0.01) (Fig. 2A). Moreover, most human leukocyte antigen (HLA) genes showed a similar expression pattern: Imm-C > Str-C > DDR-C (one-way ANOVA test, P < 0.001) (Additional file 3: Fig. S1). Furthermore, we compared the ratios of immune-stimulatory to immune-inhibitory signatures (CD8 + /CD4 + regulatory T cells) among the sarcoma subtypes. The ratios of CD8 + /CD4 + regulatory T cells were the base-2 log-transformed values of the geometric mean expression levels of all marker genes of CD8 + T cells divided by those of CD4 + regulatory T cells. Notably, the ratios exhibited similar pattern: Imm-C > Str-C > DDR-C (two-tailed Student’s *t* test, *P* < 0.05) (Fig. 2B). Taken together, these results confirmed that Imm-C had the strongest anti-tumor immune response among the three subtypes; they also demonstrated that DDR-C had the weakest anti-tumor immune response.

We also compared stromal signatures among the sarcoma subtypes. The stromal scores by ESTIMATE [16] followed the pattern: Str-C > DDR-C and Imm-C > DDR-C (one-tailed Mann–Whitney *U* test, *P* < 0.001) (Fig. 2C). Epithelial-to-mesenchymal transition (EMT) is a representative signature for assessing tumor stromal content [27]. The TCGA-SARC data showed that EMT scores were significantly higher in Str-C than in Imm-C and DDR-C (*P* < 0.05) (Fig. 2D). These data confirmed that Str-C had the strongest stromal signature. Besides, we compared the expression levels of seven DDR genes among the sarcoma subtypes. These genes included *MSH2*, *MSH3*, *MSH6*, *MLH1*, *PMS1*, *POLD1*, and *POLE*. Notably, these genes showed significantly higher expression levels in DDR-C than in Imm-C and Str-C (*P* < 0.05) (Fig. 2E). It supported that DDR-C had the strongest DDR signature.

We further compared tumor purity among the sarcoma subtypes. We evaluated tumor purity by ESTIMATE [16], which uses a cosine function of the sum of immune and stromal scores to calculate tumor purity. Interestingly, tumor purity consistently followed the pattern: Imm-C < Str-C < DDR-C, in the three datasets (one-tailed Mann–Whitney *U* test, *P* < 0.001) (Fig. 2F). These results indicated that DDR-C was most enriched with tumor cells while Imm-C harbored the highest proportion of non-tumor cells.

### Clinical and phenotypic characteristics of the sarcoma subtypes

**Fig. 3:**
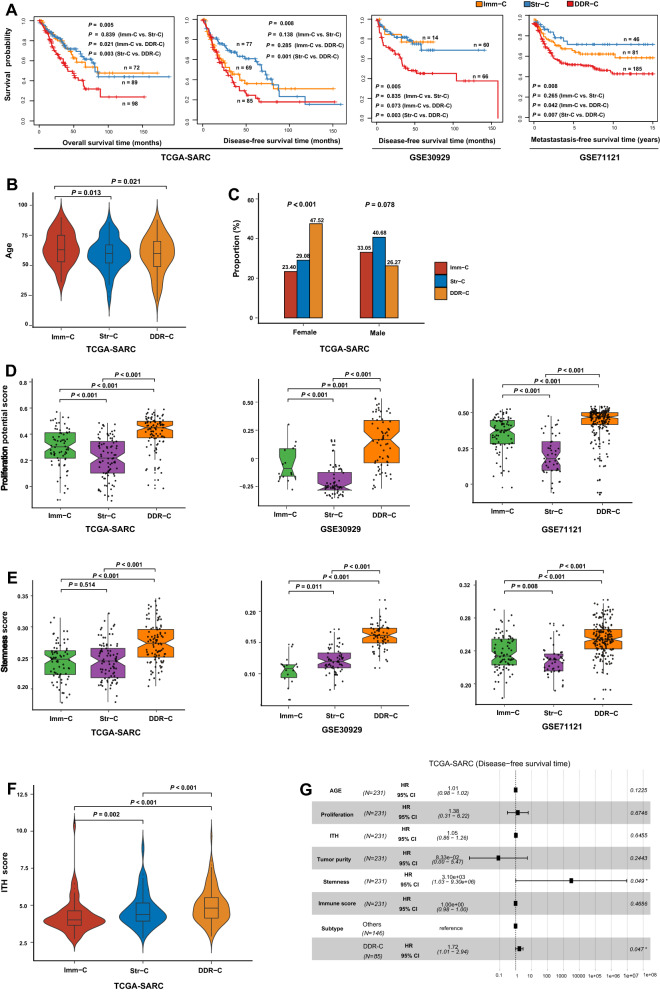
Comparisons of clinical and phenotypic features among the sarcoma subtypes. **A** Kaplan–Meier curves showing that DDR-C is likely to have the worst survival among the sarcoma subtypes. The log-rank test *P*-values are shown. **B** Comparisons of patients’ age among the sarcoma subtypes. **C** Association between gender and distribution of the sarcoma subtypes. Comparisons of proliferation potential scores (**D**), stemness scores (**E**), and intratumor heterogeneity (ITH) scores (**F**) among the sarcoma subtypes. The one-tailed Mann–Whitney *U* test *P*-values are shown in **B**, **D**, **E**, **F**. The Fisher’s exact test *P*-value and odds ratio (OR) are shown in **C**. **G** Cox proportional hazards regression analysis showing that the subtype DDR-C is a risk factor for disease-free survival prognosis in sarcomas after correcting for age, immune score, tumor purity, tumor proliferation potential, stemness, and ITH. HR, hazard ratio; CI, confidence interval

We compared survival [overall survival (OS), disease-free survival (DFS), and metastasis-free survival (MFS)] prognosis among the sarcoma subtypes. Notably, DDR-C was likely to have the worst survival consistently in the three datasets (log-rank test, *P* < 0.05), while Imm-C and Str-C showed no significant difference in survival (Fig. 3A). Interestingly, Imm-C patients were significantly older than Str-C and DDR-C patients (one-tailed Mann–Whitney *U* test, *P* < 0.05) (Fig. 3B), while Str-C and DDR-C patients had no significant difference in ages. In addition, in male patients, Str-C had the highest proportion (40.68%) and DDR-C had the lowest proportion (26.27%). In contrast, in female patients, DDR-C had the highest proportion (47.52%) and Imm-C had the lowest proportion (23.40%) (Fig. 3C).

We compared several tumor progression phenotypes among the sarcoma subtypes, including proliferation potential, stemness, and intratumor heterogeneity (ITH). These phenotypes are associated with tumor progression, immune evasion, drug resistance, and unfavorable prognosis [28, 29]. Notably, proliferation potential scores followed the pattern: Str-C < Imm-C < DDR-C, consistently in the three datasets (one-tailed Mann–Whitney *U* test, P < 0.001) (Fig. 3D). Stemness scores followed the pattern: Str-C < DDR-C and Imm-C < DDR-C (one-tailed Mann–Whitney *U* test, *P* < 0.001) (Fig. 3E). In addition, we used the DEPTH algorithm [18] to score the ITH of the sarcomas in TCGA-SARC. The ITH scores were the highest in DDR-C and the lowest in Imm-C (one-tailed Mann–Whitney U test, *P* < 0.05) (Fig. 3F). Altogether, these results confirmed that DDR-C had the worst prognosis among the three sarcoma subtypes.

Because age, immune score, tumor purity, tumor proliferation potential, stemness, and ITH are potentially associated with clinical outcomes in cancer and were significantly different among the sarcoma subtypes, the worse survival prognosis in DDR-C versus the other subtypes could have an association with these confounding variables. To explore the possibility, we performed multivariate (age, immune score, tumor purity, tumor proliferation potential, stemness, ITH, and DDR-C subtype) survival analysis by the multivariate Cox proportional hazards model. We found that the subtype DDR-C remained a significant risk factor for DFS (*P* = 0.047; hazard ratio (HR) = 1.72 and its 95% confidence interval (CI) [1.01, 2.94] (Fig. 3G) after correcting for these variables. It suggests that DDR-C is an independent risk factor for sarcomas.

### Molecular characteristics of the sarcoma subtypes

We compared various molecular characteristics among the sarcoma subtypes, including genomics, transcriptomics, methylation profiles, and proteomics. In comparisons of genomic, methylation profiles, and proteomic characteristics, we merely used the TCGA-SARC dataset since related data were not available in the other two datasets.

#### Genomic characteristics

**Fig. 4:**
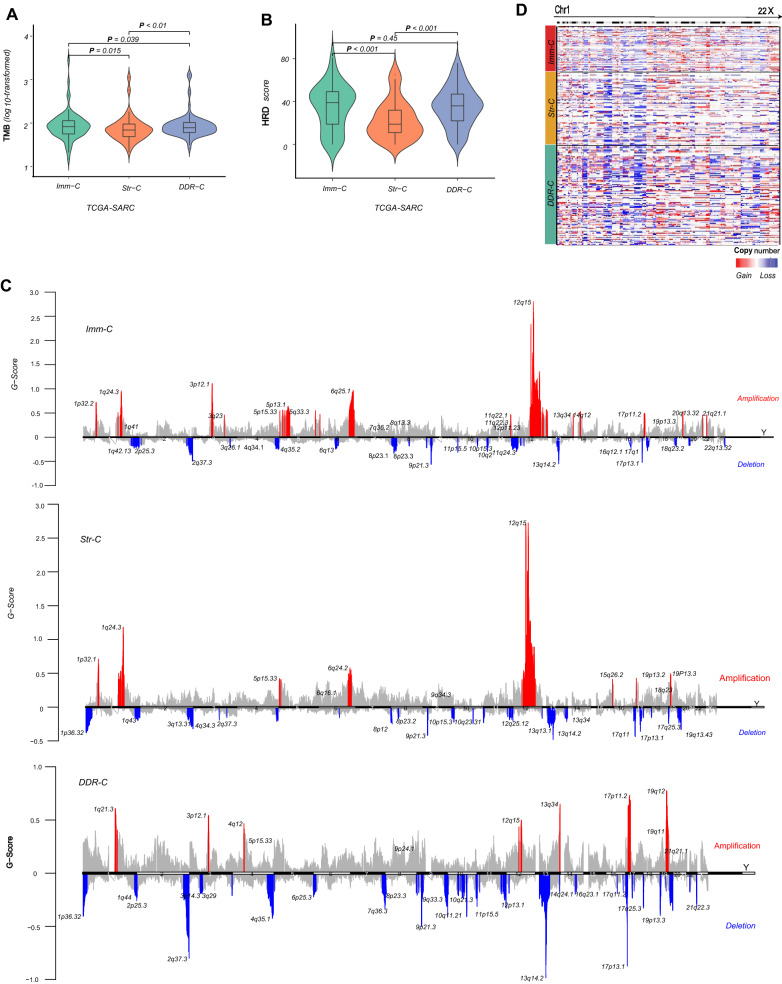
Comparisons of genomic features among the sarcoma subtypes in TCGA-SARC. Comparisons of tumor mutation burden (TMB) (**A**), homologous recombination deficiency (HRD) scores (**B**), and frequencies of arm-level copy number amplifications and deletions (**C**) among the sarcoma subtypes. **D** Copy-number heatmap of sarcomas with amplification (red) and deletion (blue) with Imm-C (top), Str-C (middle), and DDR-C (bottom). The frequencies of copy number alterations and G-scores were calculated by GISTIC2 [19]

Genomic instability often leads to a high tumor mutation burden (TMB) and/or increased CNAs [30]. We found that TMB was significantly lower in Str-C than in Imm-C and DDR-C (one-tailed Mann–Whitney *U* test, *P* < 0.05), while it showed no significant difference between Imm-C and DDR-C (Fig. 4A). Homologous recombination deficiency (HRD) may promote tumor aneuploidy levels, namely CNAs [17]. We found that HRD scores were significantly lower in Str-C than in Imm-C and DDR-C (*P* < 0.001), while they were not significantly different between Imm-C and DDR-C (Fig. 4B). In addition, we used GISTIC2 [19] to calculate arm- and focal-level somatic copy number alterations (SCNAs) and G-scores. The G-score reflects the amplitude of the SCNA and the frequency of its occurrence across a group of samples [19]. Notably, the frequencies of arm-level copy number amplifications and deletions followed the tendency: Str-C < Imm-C < DDR-C (Fig. 4C). The G-scores of copy number amplifications were significantly higher in Imm-C than in DDR-C and Str-C, while the G-scores of copy number deletions were significantly higher in DDR-C than in Imm-C and Str-C (Fig. 4D). Overall, these results indicated that Str-C was more genomically stable than Imm-C and DDR-C.

#### Somatic mutations

**Fig. 5:**
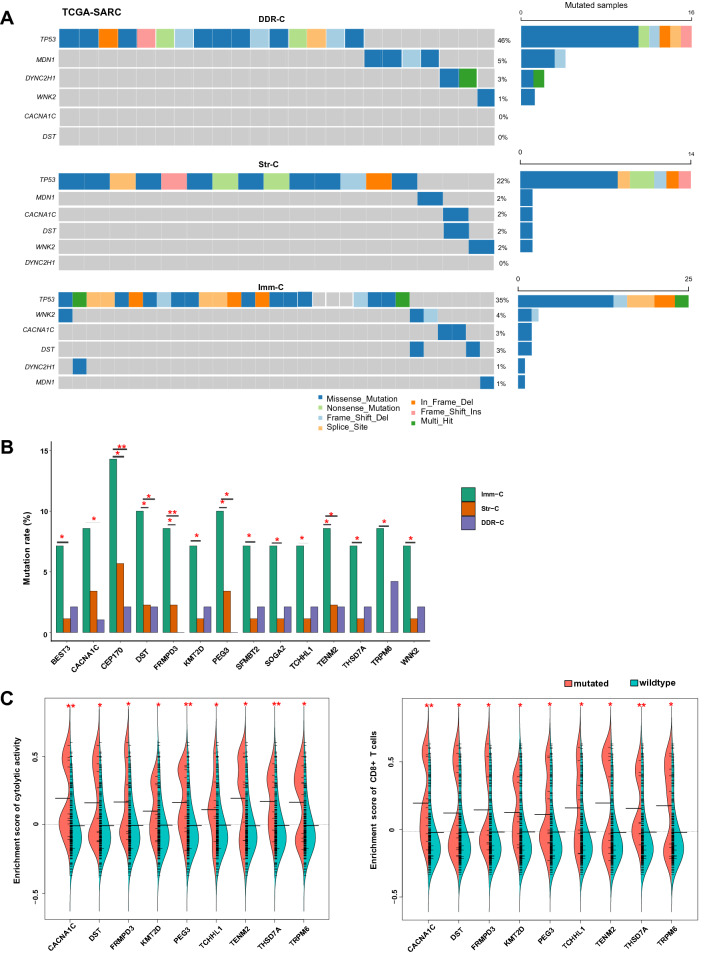
Comparisons of somatic mutation profiles among the sarcoma subtypes in TCGA-SARC. **A** 6 genes showing significantly different mutation frequencies among the sarcoma subtypes. **B** The genes showing significantly higher mutation frequencies in Imm-C than in DDR-C and Str-C (Fisher’s exact test, *P* < 0.05, OR > 4.0). *P < 0.05, **P < 0.01, ***P < 0.001. **C** The genes with higher mutation frequencies in Imm-C than in DDR-C and Str-C and their mutations associated with elevated enrichment levels of CD8 + T cells and/or immune cytolytic activity (one-tailed Mann–Whitney *U* test, *P* < 0.05)

We observed that *TP53* had a significantly higher mutation frequency in DDR-C than in Imm-C and Str-C (Fisher’s exact test, *P* = 0.01, odds ratio (OR) = 2.0) (Fig. 5A). It could partially explain why DDR-C had the worst prognosis among the three subtypes since *TP53* mutations have been associated with unfavorable outcomes in various cancers [31]. Indeed, we found that *TP53*-mutated tumors had a significant worse OS and DFS than *TP53*-wildtype tumors in DDR-C (*P* < 0.02). On the other hand, *TP53* mutations were less frequent in Str-C than in DDR-C and Imm-C (*P* = 0.009, OR = 0.46). This result could explain why Str-C was more genomically stable than the other subtypes because p53 plays an important role in the maintenance of genomic stability [32]. In addition, *DYNC2H1* and *MDN1* were more frequently mutated in DDR-C than in Imm-C and Str-C (*P* = 0.04, OR = 4.14) (Fig. 5A). There were 14 genes showing significantly higher mutation frequencies in Imm-C than in DDR-C and Str-C (*P* < 0.05, OR > 4.0) (Fig. 5B). These genes included *BEST3*, *CACNA1C*, *CEP170*, *DST*, *FRMPD3*, *KMT2D*, *PEG3*, *SFMBT2*, *SOGA2*, *TCHHL1*, *TENM2*, *THSD7A*, *TRPM6*, and *WNK2*. Notably, the mutations in many of these genes were positively correlated with the enrichment scores of CD8 + T cells and/or immune cytolytic activity, including *CACNA1C*, *THSD7A*, *PEG3*, *TENM2*, *KMT2D*, *TRPM6*, *FRMPD3*, *DST*, and *TCHHL1* (*P* < 0.05) (Fig. 5C). It is consistent with the strongest immune signatures presented in Imm-C.

#### DNA methylation

**Fig. 6:**
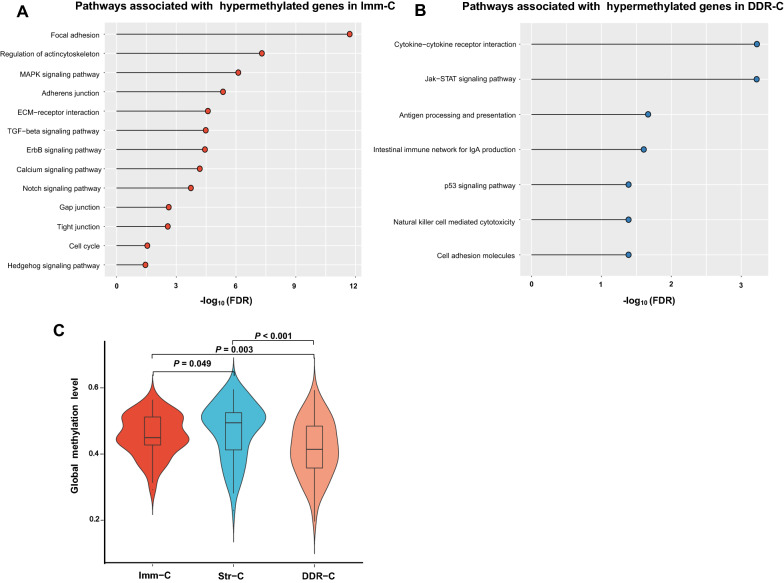
Comparisons of DNA methylation profiles among the sarcoma subtypes in TCGA-SARC. **A** The KEGG pathways significantly associated with 1716 hypermethylated genes in Imm-C. **B** The KEGG pathways significantly associated with 180 hypermethylated genes in DDR-C. **C** Comparisons of global methylation levels among the sarcoma subtypes. The data of global methylation levels were obtained from the publication by Jung et al. [33]. In **A**, **B**, the KEGG pathways were identified by GSEA [15]

We found 1716 genes showing significantly higher methylation levels in Imm-C compared to both Str-C and DDR-C [one-tailed Mann–Whitney U test, false discovery rate (FDR) < 0.05]. In contrast, 124 and 180 genes had significantly higher methylation levels in Str-C and DDR-C, respectively, compared to other subtypes. Notably, most of these genes showed significant inverse correlations of their expression levels with methylation levels (Pearson correlation, *P* < 0.05) (Additional file 4: Table S3). The top 30 genes with the most significant upregulation of methylation levels in each of the three subtypes are presented Additional file 5: Fig. S2. The KEGG pathways significantly associated with the 1716 hypermethylated genes in Imm-C mainly included focal adhesion, regulation of actin cytoskeleton, MAPK signaling, adherens junction, ECM-receptor interaction, TGF-β, ErbB signaling, calcium signaling, Notch signaling, gap junction, tight junction, cell cycle, and Hedgehog signaling (Fig. 6A). These results supported that Imm-C had lower activities of stromal signatures and oncogenic pathways. In contrast, the pathways associated with the 180 hypermethylated genes in DDR-C mainly included cytokine-cytokine receptor interaction, Jak-STAT signaling, antigen processing and presentation, the intestinal immune network for IgA production, natural killer cell-mediated cytotoxicity, cell adhesion molecules, and p53 signaling. These results confirmed that DDR-C had a lower anti-tumor immune response and p53 function (Fig. 6B). It has been shown that low methylation levels correlate with increased TMB and CNAs in cancer [33]. We compared global methylation levels [33] among the sarcoma subtypes and observed the pattern: Str-C > Imm-C > DDR-C (*P* < 0.05) (Fig. 6C). This conforms with our previous results showing that Str-C had the lowest TMB and CNAs among the sarcoma subtypes.

#### Protein expression profiles

**Fig. 7:**
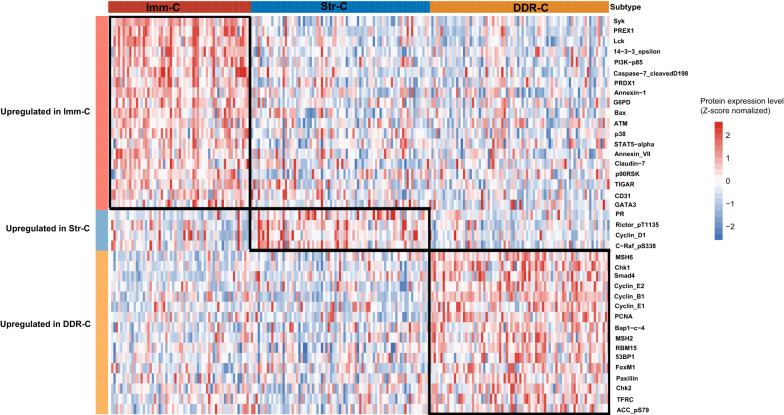
Comparisons of protein expression profiles among the sarcoma subtypes in TCGA-SARC. Heat-map showing the proteins with significantly higher expression levels in a sarcoma subtype than in other sarcoma subtypes (two-tailed Student’s *t* test, FDR < 0.05)

We compared the expression levels of 192 proteins among the sarcoma subtypes using the TCGA protein expression profiling data. We found 19 proteins having significantly higher expression levels in Imm-C than in both Str-C and DDR-C (two-tailed Student’s *t* test, FDR < 0.05). These proteins included Syk, PREX1, Lck, 14-3-3_epsilon, PI3K-p85, Caspase-7_cleavedD198, PRDX1, Annexin-1, G6PD, Bax, ATM, p38, STAT5-alpha, Annexin_VII, Claudin-7, p90RSK, TIGAR, CD31, and GATA3 (Fig. 7). As expected, these proteins displayed significant positive correlations of expression levels with anti-tumor immune signature scores (Additional file 6: Fig. S3). In fact, many of these proteins are involved in immune regulation. For example, Syk plays a crucial role in adaptive and innate immune regulation [34]. PREX1 is a key regulator of neutrophil function [35]. Lck has a crucial role in T-cell development and activation [36]. Annexin-1 is an anti-inflammatory protein that can drive hyperactivation of T cells during pathological conditions [37]. CD31 is an adhesion molecule expressed in various immune cells [38]. GATA3 is a transcription factor essential for regulating the function of human type 2 innate lymphoid cells [39]. STAT5 is important in the maintenance of immune function [40].

16 proteins displayed significantly higher expression levels in DDR-C than in both Str-C and Imm-C (FDR < 0.05) (Fig. 7). These proteins included MSH6, Chk1, Smad4, Cyclin_E2, Cyclin_B1, Cyclin_E1, PCNA, Bap1-c-4, MSH2, RBM15, 53BP1, FoxM1, Paxillin, Chk2, TFRC, and ACC_pS79. Among these proteins, MSH2, MSH6, PCNA, 53BP1, and RBM15 are involved in DNA damage repair, consistent with the strongest DDR activity in DDR-C. Besides, many of these proteins are involved in cell cycle regulation, including Chk1, Chk2, Cyclin_B1, Cyclin_E1, Cyclin_E2, FoxM1, Bap1-c-4. Again, it is consistent with the strongest cell cycle activity presented in DDR-C. In addition, four proteins showed significantly higher expression levels in Str-C than in both DDR-C and Imm-C (FDR < 0.05), including PR, Rictor_pT1135, Cyclin_D1, and C-Raf_pS338 (Fig. 7)*.*

### A risk scoring model based on the expression levels of five genes in the pathways

**Fig. 8:**
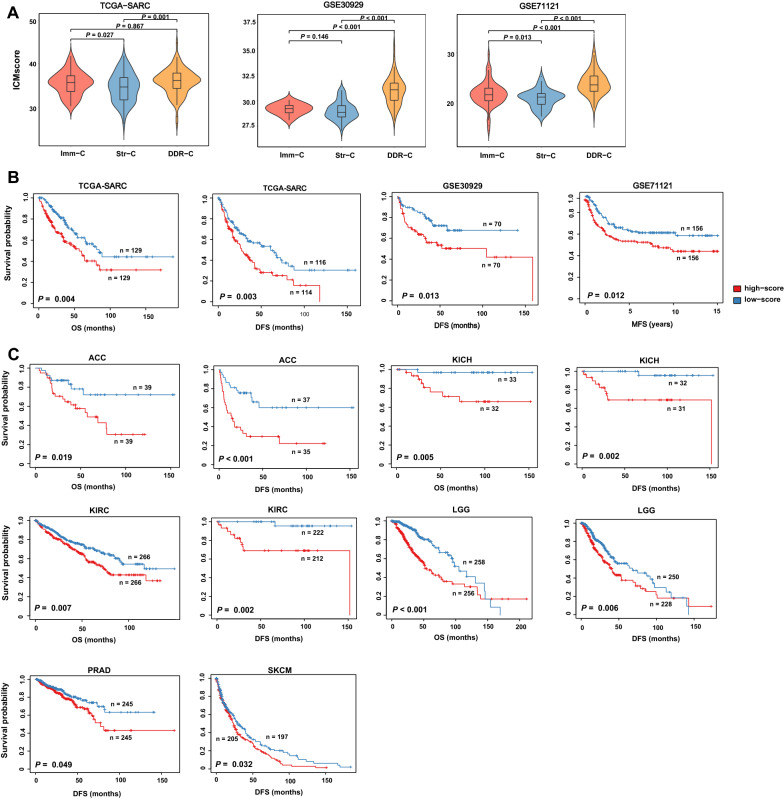
Associations of the prognostic risk score (ICMScore) with the sarcoma subtypes and survival prognosis in sarcomas and other cancer types. **A** Comparisons of ICMScores among sarcoma subtypes. Kaplan–Meier curves showing that higher-ICMScore (> median) tumors have worse survival prognosis than lower-ICMScore (< median) tumors in sarcomas (**B**) and in six other cancer types (**C**). The log-rank test *P*-values are shown. ICMScore is a linear risk scoring model for evaluating the prognostic risk of sarcomas. It was developed based on the expression levels of five genes in five of the 14 pathways for clustering analysis, including T cell receptor signaling (*CD40LG*), cell cycle (*CDC25A*), mismatch repair (*MSH2*), focal adhesion (*FLT4*), and calcium signaling (*ADCY2*). OS, overall survival; DFS, disease-free survival; MFS, metastasis-free survival; ACC, adrenocortical carcinoma; KICH, kidney chromophobe; KIRC, kidney renal clear cell carcinoma; LGG, brain lower grade glioma; PRAD, prostate adenocarcinoma; SKCM, skin cutaneous melanoma

Using the TCGA-SARC dataset, we developed a linear risk scoring model (ICMScore) to evaluate the prognostic risk of sarcomas based on the expression levels of five genes, including *CD40LG*, *CDC25A*, *MSH2*, *FLT4*, and *ADCY2*. The five genes were involved in five of the 14 pathways for clustering analysis, including T cell receptor signaling (*CD40LG*), cell cycle (*CDC25A*), mismatch repair (*MSH2*), focal adhesion (*FLT4*), and calcium signaling (*ADCY2*). ICMScore calculates risk score in a tumor as follows: ICMScore = 0.76 × exp(*CD40LG*) + 0.73 × exp(*FLT4*) − 0.20 × exp(*ADCY2*) + 1.97 × exp(*CDC25A*) + 1.50 × exp(*MSH2*), where exp(*X*) denotes the expression level of gene *X* in the tumor sample. To prove that ICMScore is an authentic risk factor in sarcomas, we compared ICMScores among the three sarcoma subtypes and analyzed their correlation with survival prognosis in sarcomas. In the three sarcoma datasets, As expected, ICMScores was significantly higher in DDR-C than in Imm-C and Str-C (two-tailed Student’s t test, P < 0.05) (Fig. 8A). Survival analyses showed that higher-ICMScore (> median) tumors had significantly worse survival (OS, DFS, and MFS) prognosis than lower-ICMScore (< median) tumors in the sarcoma datasets (log-rank test, *P* < 0.05) (Fig. 8B). These results supported that ICMScore was a prognostic risk factor in sarcomas. Interestingly, we found that ICMScore was also a prognostic risk factor in many other cancer types, including adrenocortical carcinoma (ACC), kidney chromophobe (KICH), kidney renal clear cell carcinoma (KIRC), brain lower grade glioma (LGG), prostate adenocarcinoma (PRAD), and skin cutaneous melanoma (SKCM), as evidenced by that elevated ICMScores were associated with worse OS and/or DFS in these TCGA cancer types (Fig. 8C).

### Validation by analyzing scRNA-seq data

**Fig. 9:**
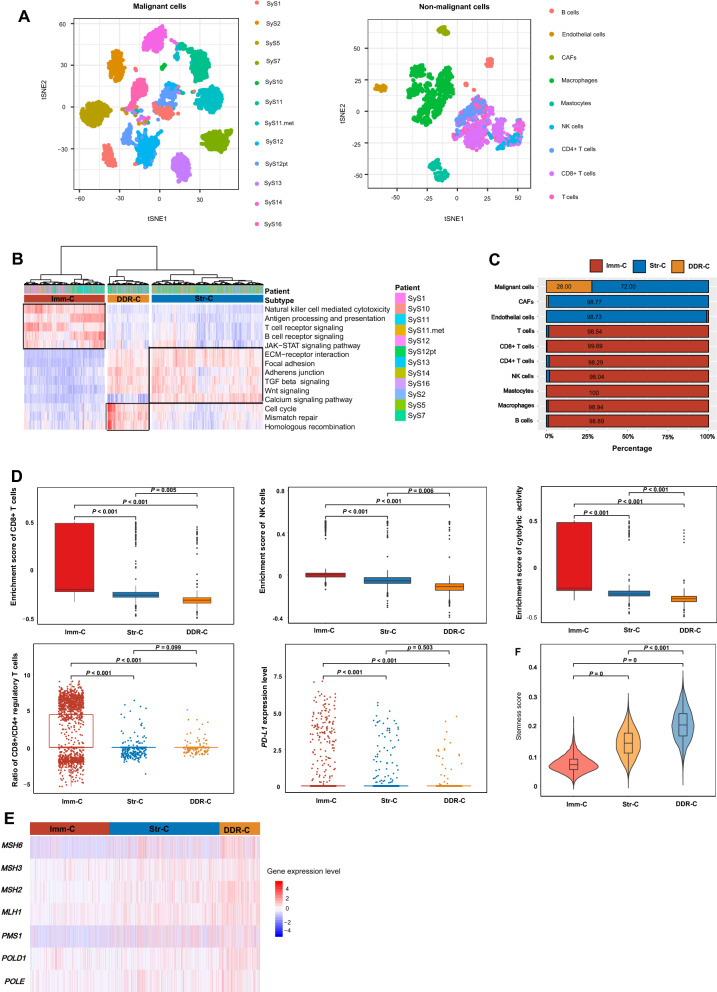
Validation of the pathway-based clustering method in a single cell RNA-Seq (scRNA-seq) dataset. **A** Clustering of 4371 malignant cells and 2580 non-malignant cells by the tSNE algorithm [21]. **B** Hierarchical clustering based on the enrichment scores of the 14 pathways identifies three subtypes of 6951 single cells from 12 sarcoma patients. **C** Distribution of the 6951 single cells in the three subtypes. Comparisons of immune signature scores (**D**), expression levels of DDR genes (**E**), and stemness scores (**F**) among the three subtypes

Using the pathway-based clustering method, we analyzed a scRNA-seq dataset (GSE131309), which involved gene expression profiles in 6951 single cells from 12 sarcoma [advanced synovial sarcoma (SyS)] patients. The 6951 single cells included 4371 tumor cells, 90 B cells, 943 macrophages, 185 mastocytes, 102 NK cells, 235 CD4 + T cells, 659 CD8 + T cells, 206 T cells, 79 endothelial cells, and 81 cancer-associated fibroblasts (CAFs). Using the t-distributed stochastic neighbor embedding (tSNE) algorithm [21], we clustered 4371 malignant (tumor) cells and 2580 non-malignant cells, respectively (Fig. 9A). The malignant cells from 12 different patients were clearly separated. Meanwhile, the non-malignant cells were clustered into nine different groups, namely B cells, macrophages, mastocytes, NK cells, CD4 + T cells, CD8 + T cells, T cells, endothelial cells, and CAFs.

We performed hierarchical clustering of the 6951 single cells based on the enrichment scores of the 14 pathways identified three subtypes of these cells (Fig. 9B). Notably, almost all immune cells (B cells, macrophages, mastocytes, NK cells, CD4 + T cells, CD8 + T cells, and T cells) were included in Imm-C, and all stromal cells (endothelial cells and CAFs) were included in Str-C (Fig. 9C). The 4371 tumor cells were classified into Str-C (n = 3147) and DDR-C (n = 1224). These results support the stability and reliability of our method.

In the scRNA-seq dataset, the enrichment scores of anti-tumor immune signatures (CD8 + T cells, NK cells, and immune cytolytic activity) were significantly higher in Imm-C than in Str-C and DDR-C (Fig. 9D). The ratios of CD8 + /CD4 + regulatory T cells were also significantly higher in Imm-C than in Str-C and DDR-C (Fig. 9D). Interestingly, *PD-L1* expression levels were significantly higher in Imm-C than in Str-C and DDR-C (Fig. 9D). It indicates that PD-L1 is more abundant in immune cells than in stromal and tumor cells. The seven DDR genes (*MSH2*, *MSH3*, *MSH6*, *MLH1*, *PMS1*, *POLD1*, and *POLE*) exhibited the consistent expression pattern: Imm-C < Str-C < DDR-C (two-tailed Student’s *t* test, *P* < 0.001) (Fig. 9E), supporting that DDR-C has the strongest DDR activity. Likewise, stemness scores exhibited the pattern: Imm-C < Str-C < DDR-C (one-tailed Mann–Whitney *U* test, *P* < 0.001) (Fig. 9F). It is consistent with our previous results of the highest stemness scores shown in DDR-C in bulk tumors. Overall, the results in the scRNA-seq dataset are in line with the results in the bulk tumors.

## Discussion

In this study, we proposed a novel classification method for sarcomas based on the enrichment scores of 14 pathways, which were involved in immune, stromal, DDR, and oncogenic signatures. In three datasets for bulk tumors and a scRNA-seq dataset, we reproducibly identified three sarcoma subtypes: Imm-C, Str-C, and DDR-C. Imm-C had the strongest anti-tumor immune signatures and the lowest ITH; Str-C showed the strongest stromal signatures, the highest genomic stability and global methylation levels, and the lowest proliferation potential; DDR-C had the highest DDR activity, expression of the cell cycle pathway, tumor purity, stemness scores, proliferation potential, and ITH, the most frequent *TP53* mutations, and the worst survival. It is interesting to observe that there was no significantly different TMB between DDR-C and Imm-C, while their immune infiltration levels were significantly different. Two possible reasons could explain this observation: (1) DDR-C had more frequent arm-level copy number amplifications and deletions than Imm-C; and (2) DDR-C had higher ITH than Imm-C, since both CNAs [41] and ITH [18] are negatively correlated with anti-tumor immune response. DDR-C displayed worse clinical outcomes than Imm-C and Str-C. It could be attributed to the high proliferation potential, stemness, ITH, and genomic instability in DDR-C. Meanwhile, the lowest ratios of immune-stimulatory/immune-inhibitory signatures in DDR-C indicate the least activation of anti-tumor immune response in this subtype. It could also be a factor that leads to the worst prognosis in DDR.

The TCGA Research Network analyzed six types of adult soft tissue sarcomas: DDLPS, LMS, UPS, MFS, MPNST, and SS [1]. We found that 52%, 38%, and 10% of DDLPS tumors belonged to Str-C, Imm-C, and DDR-C, respectively (Additional file 7: Fig. S4A). It indicates that most DDLPS tumors are Str-C or Imm-C. In contrast, 80% of SS and 67% of ULMS tumors are DDR-C, indicating that SS and ULMS are dominated by DDR-C. In addition, 0%, 7%, and 8% of SS, ULMS, and STLMS tumors belonged to Imm-C, compared to 55% of UPS tumors being Imm-C; 5% of UPS tumors were Str-C, compared to 52% of DDLPS and 53% of STLMS tumors belonging to Str-C. Taken together, these data suggest that the different types of adult soft tissue sarcomas are dominated by different subtypes we identified. In another study [42], Thorsson et al. identified six immune subtypes of TCGA pan-cancer, including wound healing, IFN-γ dominant, inflammatory, lymphocyte depleted, immunologically quiet, and TGF-β dominant. We found that the wound healing sarcomas were mainly Str-C and DDR-C, and only 11% were Imm-C; the IFN-γ dominant sarcomas were mainly Imm-C and DDR-C, and only 16% were Str-C; the inflammatory sarcomas were dominated by Str-C (71%), compared to 10% of the lymphocyte depleted tumors were Str-C (Additional file 7: Fig. S4B). This study revealed that the inflammatory subtype and lymphocyte depleted subtype had the best and worst prognosis, respectively. It is in accord with our results of the worst survival in DDR-C in that 10% of the inflammatory sarcomas and 61% of the lymphocyte depleted sarcomas belonged to DDR-C, respectively. In addition, Gibault et al. identified five subtypes (Clusters A–E) of soft tissue sarcomas based on gene expression profiles [6]. Cluster B was a subgroup of sarcomas with a favorable metastasis outcome in multivariate analysis [6], which constituted the least proportion (9%) of DDR-C among the five clusters (Additional file 7: Fig. S4C). It is consistent with the worst prognosis of DDR-C among the three subtypes we identified. Clusters C–E harbored poorly differentiated LMS, UPS, MFS, and pleiomorphic sarcomas of the limbs and displayed combinations of expression of genes involved in invasion, extracellular matrix, or inflammatory processes, which were predominant in DDR-C, Str-C, and Imm-C, respectively. Hence, our subtyping method demonstrated a clearer separation of sarcomas with respect to pathways and biological processes compared to previous subtyping methods.

## Conclusions

Based on the enrichment scores of 14 pathways associated with immune, stromal, and DDR signatures, we classified sarcomas into three subtypes. The three sarcoma subtypes were characterized by different immune infiltration levels, stromal signatures, DDR activity, genome features, tumor progression phenotypes, and clinical outcomes. Our new classification method for sarcomas provides novel insights into tumor biology and clinical implications for this disease.

## Supplementary Information

**Additional file 1: Table S1.** A summary of the datasets analyzed.

**Additional file 2: Table S2**. Pathways, immune signatures, and biological processes and their marker genes.

**Additional file 3: Fig. S1.** Comparisons of the expression levels of human leukocyte antigen (HLA) genes among the sarcoma subtypes. The one-way ANOVA test *P*-values are shown. * *P* < 0.05, ** *P* < 0.01, *** *P* < 0.001.

**Additional file 4: Table S3.** Pearson correlations between the methylation levels and expression levels of the genes having significantly different methylation levels among the sarcoma subtypes.

**Additional file 5: Fig. S2.** Heatmap showing top 30 genes with the most significant upregulation of methylation levels in each of the three subtypes.

**Additional file 6: Fig. S3.** Positive correlations between the expression levels of 19 proteins significantly upregulated in Imm-C and immune signature scores in TCGA-SARC. * *P* < 0.05, ** *P* < 0.01, *** *P* < 0.001.

**Additional file 7: Fig. S4.** Overlaps between our subtyping and other subtyping of sarcoma in TCGA-SARC. (A) Proportions of our subtypes in six types of adult soft tissue sarcomas: DDLPS, LMS (ULMS and STLMS), UPS, MFS, MPNST, and SS. (B) Proportions of our subtypes in the immune subtypes of sarcomas. (C) Proportions of the five subgroups (Clusters A-E) in our subtypes.

## Data Availability

The data supporting the conclusions of this article were presented in Additional Tables.

## Code availability

We performed all computational and statistical analyses in the R programming environment (version 4.1.0, https://cran.r-project.org/).
