# Supplementary figures and images for: Subtyping of sarcomas based on pathway enrichment scores in bulk and single cell transcriptomes
Source: J Transl Med. 2022 Jan 29;20:48. doi: 10.1186/s12967-022-03248-3 (PMC8800234; doi:10.1186/s12967-022-03248-3)

Fig. S1

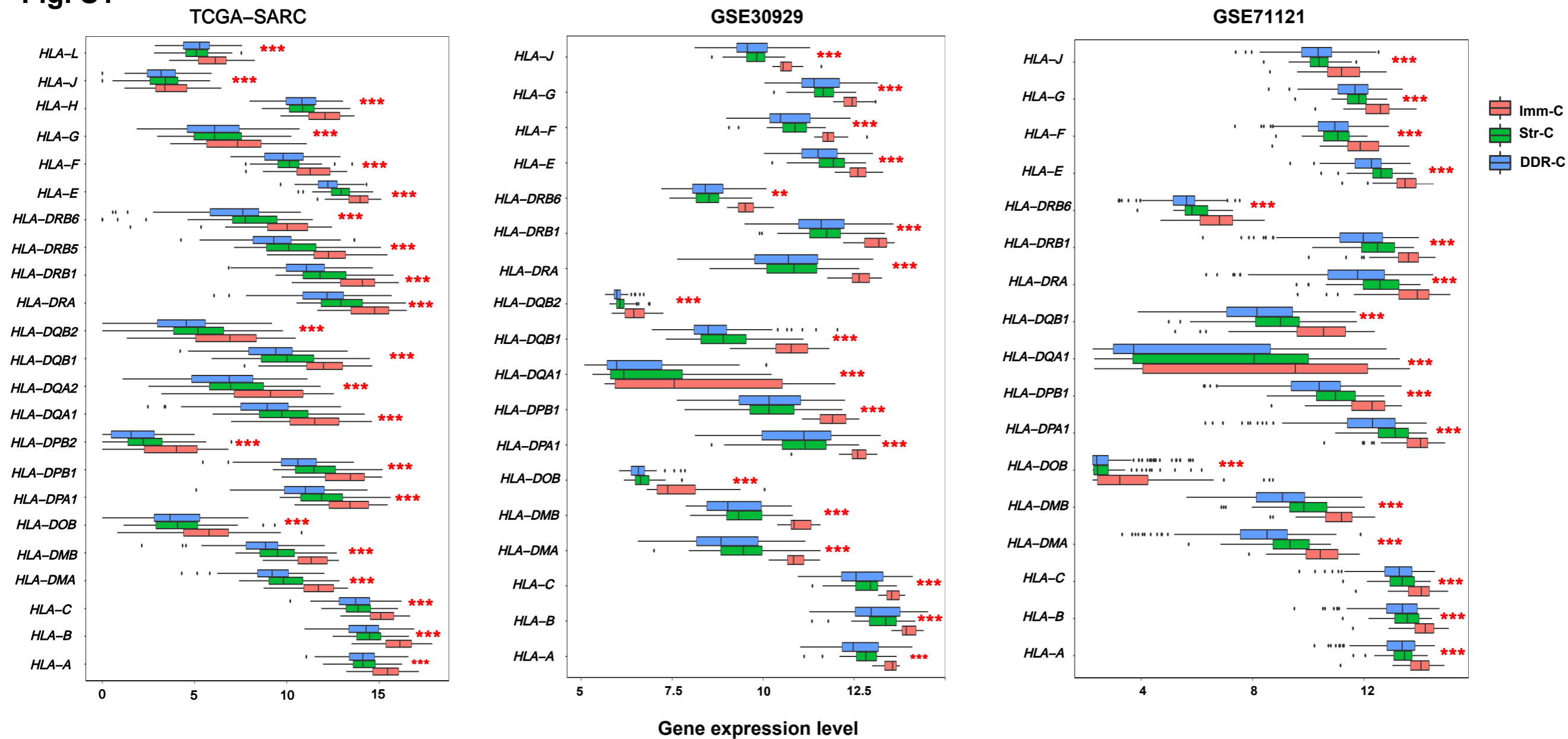

Supplement: Supplementary file 3 — Additional file 3: Fig. S1. Comparisons of the expression levels of human leukocyte antigen (HLA) genes among the sarcoma subtypes. The one-way ANOVA test P-values are shown. * P < 0.05, ** P < 0.01, *** P < 0.001. [file 12967_2022_3248_MOESM3_ESM.pdf]

Fig. S2

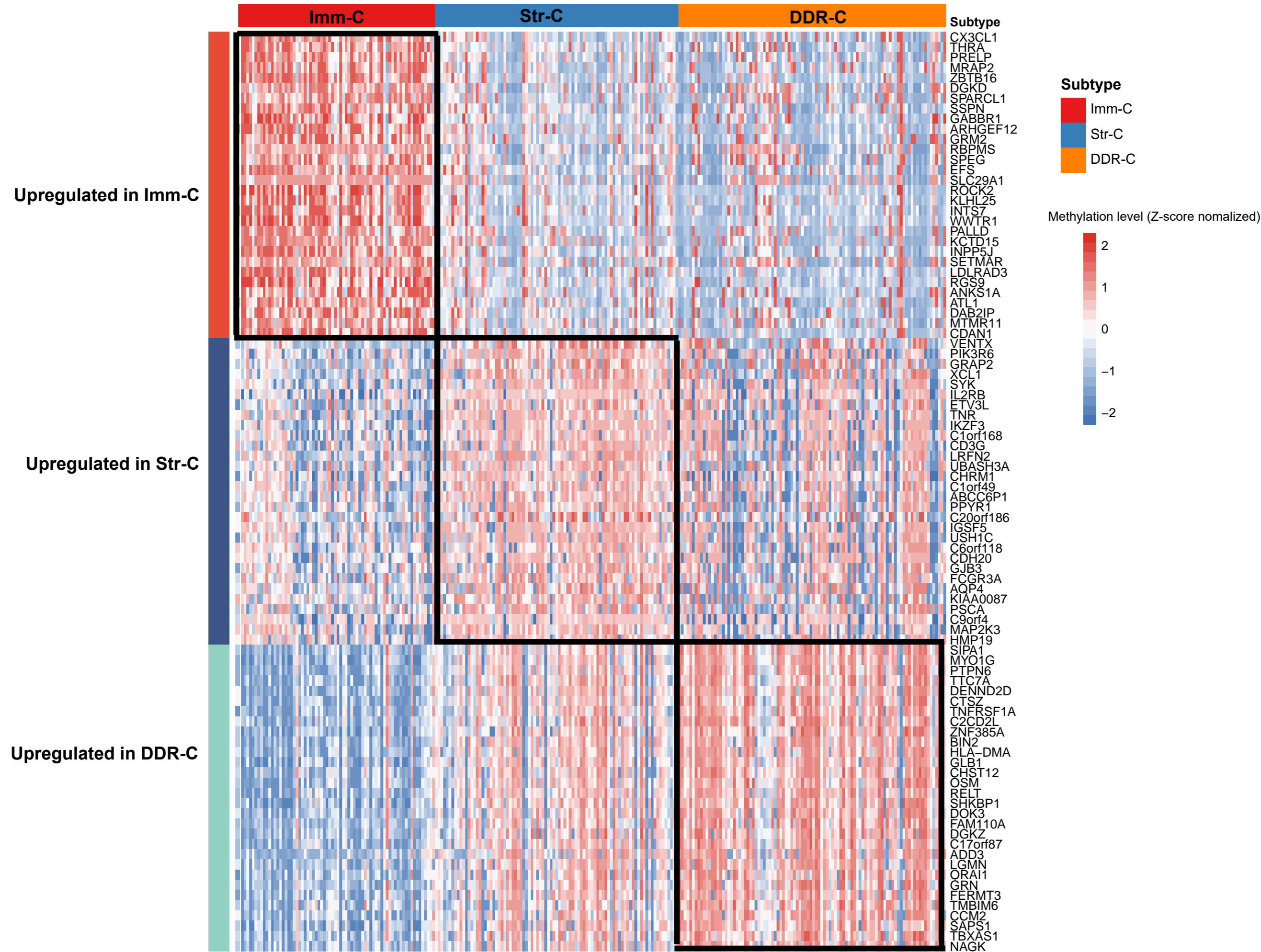

Supplement: Supplementary file 5 — Additional file 5: Fig. S2. Heatmap showing top 30 genes with the most significant upregulation of methylation levels in each of the three subtypes. [file 12967_2022_3248_MOESM5_ESM.pdf]

Fig. S3

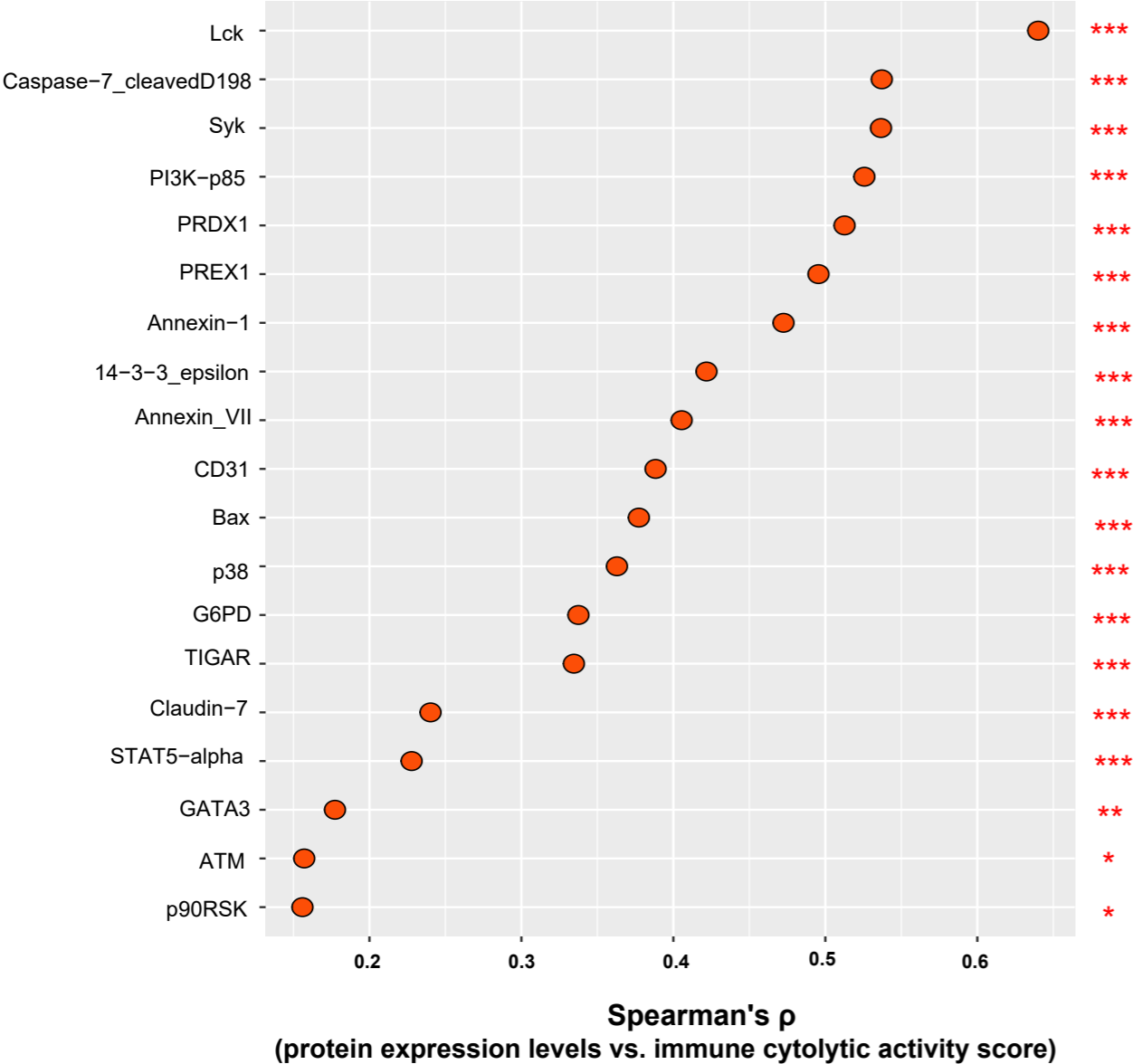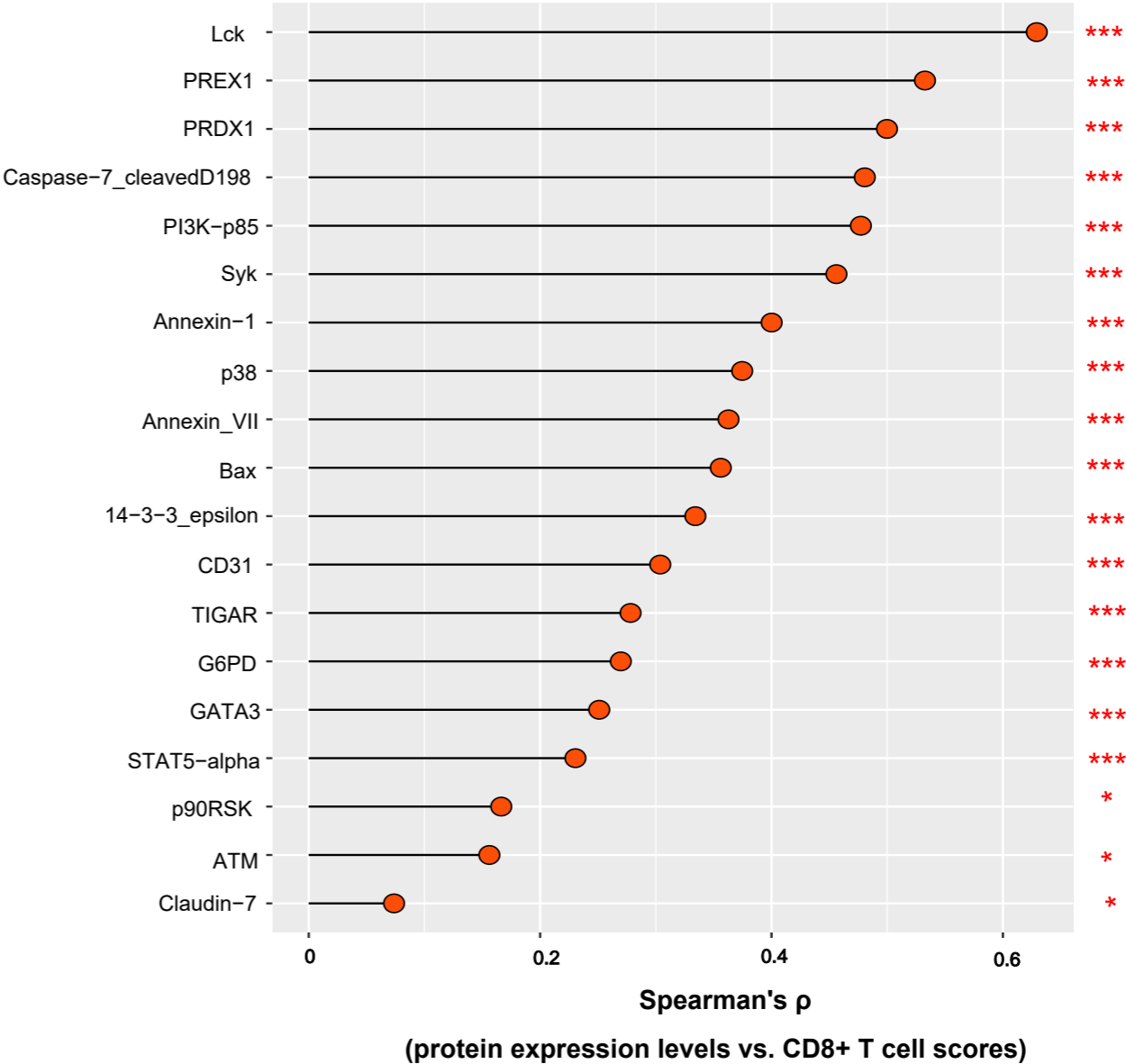

Supplement: Supplementary file 6 — Additional file 6: Fig. S3. Positive correlations between the expression levels of 19 proteins significantly upregulated in Imm-C and immune signature scores in TCGA-SARC. * P < 0.05, ** P < 0.01, *** P < 0.001. [file 12967_2022_3248_MOESM6_ESM.pdf]

Fig. S4

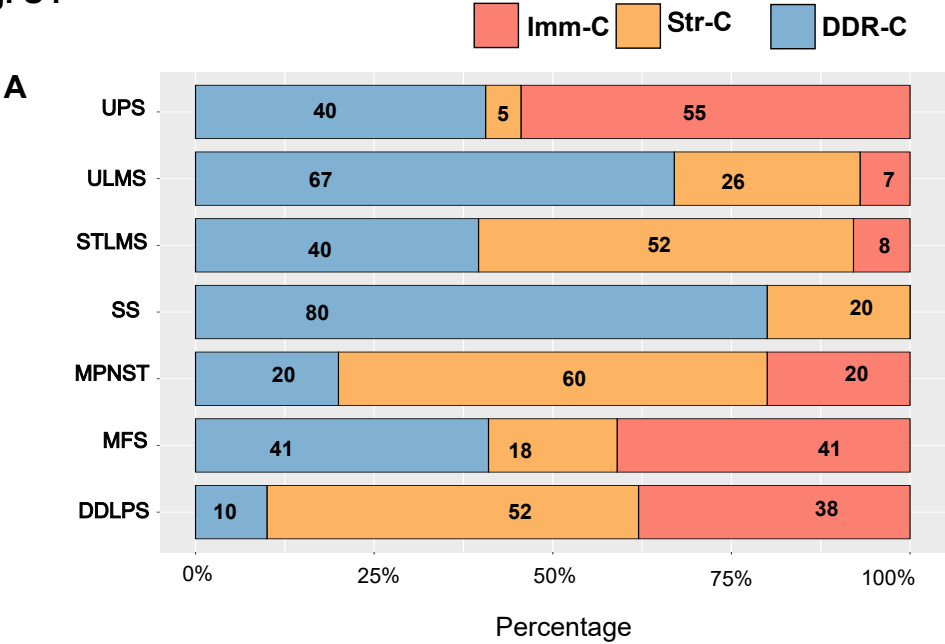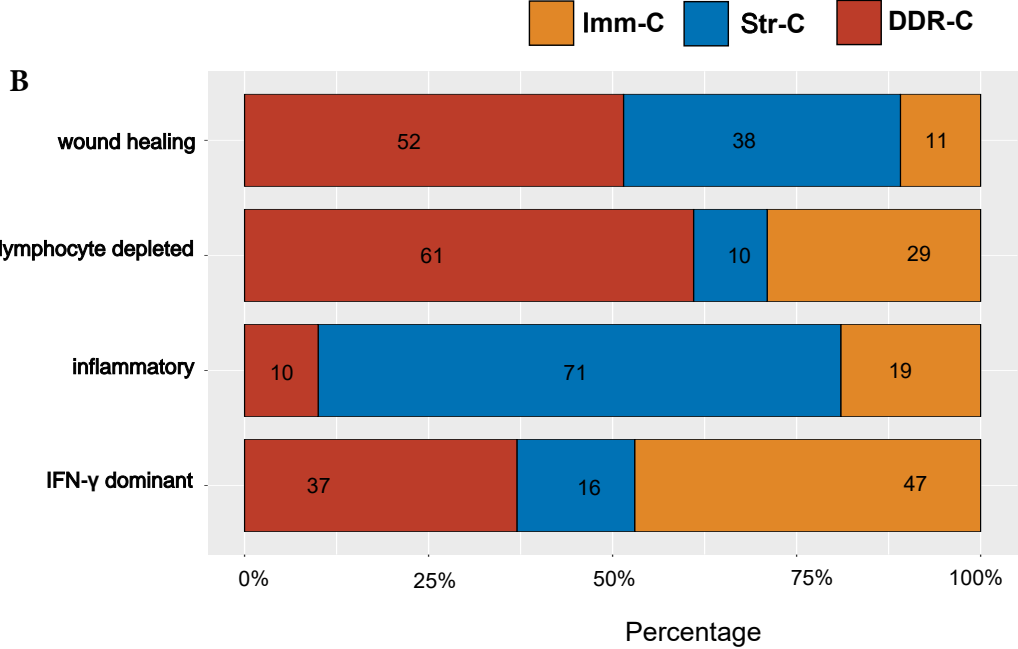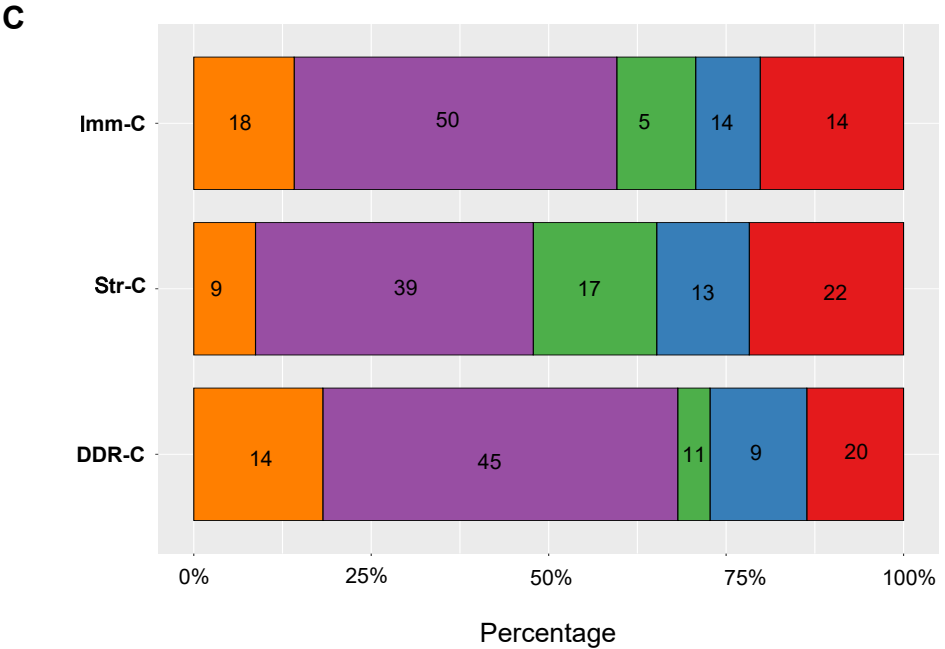

Supplement: Supplementary file 7 — Additional file 7: Fig. S4. Overlaps between our subtyping and other subtyping of sarcoma in TCGA-SARC. (A) Proportions of our subtypes in six types of adult soft tissue sarcomas: DDLPS, LMS (ULMS and STLMS), UPS, MFS, MPNST, and SS. (B) Proportions of our subtypes in the immune subtypes of sarcomas. (C) Proportions of the five subgroups (Clusters A-E) in our subtypes. [file 12967_2022_3248_MOESM7_ESM.pdf]
